# Interpreting intracorporeal landscapes: how patients visualize pathophysiology and utilize medical images in their understanding of chronic musculoskeletal illness

**DOI:** 10.1080/09638288.2018.1443162

**Published:** 2018-02-26

**Authors:** Andrew J. Moore, Jane C. Richardson, Miriam Bernard, Julius Sim

**Affiliations:** a Musculoskeletal Research Unit, Bristol Medical School, University of Bristol, Bristol, UK;; b Arthritis Research UK Primary Care Centre, Research Institute for Primary Care and Health Sciences, Keele University, Staffordshire, UK;; c School of Social Science and Public Policy, Keele University, Staffordshire, UK

**Keywords:** Pathophysiology, pain, practitioner–patient communication, patient information, osteoarthritis

## Abstract

Medical science and other sources, such as the media, increasingly inform the general public’s understanding of disease. There is often discordance between this understanding and the diagnostic interpretations of health care practitioners (HCPs). In this paper – based on a supra-analysis of qualitative interview data from two studies of joint pain, including osteoarthritis – we investigate how people imagine and make sense of the pathophysiology of their illness, and how these understandings may affect self-management behavior. We then explore how HCPs’ use of medical images and models can inform patients’ understanding. In conceptualizing their illness to make sense of their experience of the disease, individuals often used visualizations of their inner body; these images may arise from their own lay understanding, or may be based on images provided by HCPs. When HCPs used anatomical models or medical images judiciously, patients’ orientation to their illness changed. Including patients in a more collaborative diagnostic event that uses medical images and visual models to support explanations about their condition may help them to achieve a more meaningful understanding of their illness and to manage their condition more effectively.

**Implications for Rehabilitation**
- Chronic musculoskeletal pain is a leading cause of pain and years lived with disability, and despite its being common, patients and healthcare professionals often have a different understanding of the underlying disease.
- An individual’s understanding of his or her pathophysiology plays an important role in making sense of painful joint conditions and in decision-making about self-management and care.
- Including patients in a more collaborative diagnostic event using medical images and anatomical models to support explanations about their symptoms may help them to better understand their condition and manage it more effectively.
- Using visually informed explanations and anatomical models may also help to reassure patients about the safety and effectiveness of core treatments such as physical exercise and thereby help restore or improve patients’ activity levels and return to social participation.

## Introduction

Life…is a constant process of negotiating landscapes (internal and external), and interpretation is as necessary to the process as is breathing, no matter how bizarre or fantastic it may seem from the outside looking in. [1, p.509]

In an attempt to make sense of illness, people employ narrative descriptions to give meaning to that which is invisible, such as painful sensations or the internal landscapes of the body. However, Scarry [2] notes that the experience of pain is particularly resistant to language and objective expression, and its essential subjectivity makes it largely “unshareable” with others [3,4]. This reflects the private nature of pain:

As a mode of subjectivity, pain is intensely private… my pain is radically my own. There exists no objectification of it that would allow another to share my pain. [4, p.186]

Hydén [5, p.264] argues that attempts to communicate pain verbally are further hindered by the lack of “a standard set of descriptive terms for various types of pain.” Individuals therefore need to enlist auxiliary linguistic strategies, using metaphors or paralinguistic modes of communication such as gesture or facial expression – though van Hooft [4] suggests that these paralinguistic means of expression may become repressed, and therefore less effective, in pain that has become chronic. Similarly, attempts on the part of practitioners to objectify pain, by standardizing the way in which it is measured, led to the development of visual analog scales (VASs), normally representing a scale numbered from 0 to 10 [6]. However, it has also long been recognized that the subjective nature of pain is poorly represented by VAS scales [7], on which scores may vary as much as 20% on repeated testing [8]. Furthermore, the VAS may not be responsive to various types of pain [9].

Nonetheless, despite pain’s resistance to the resources of language [2,10], people persevere in finding ways to convey what they feel. This becomes evident when they describe what they think is happening *beneath* their skin, *inside* their bodies, through imaginings based on some knowledge of anatomy, or through comparisons with everyday observations of their life world. Thus, Padfield [11, p.242] suggests that an understanding of painful conditions requires the use of both language and image, and that “we are forced to mediate language via the image and vice versa to unravel enough meaning to arrive at a shared understanding”. It is this use of visualization, imagination and symbolic images in language that we explore here in relation to patients’ descriptions of musculoskeletal conditions.

We begin this exploration by theorizing about the role of the image in explanations of illness. Specifically, we examine the relationship between, on the one hand, what we call the “intracorporeal landscape” (the physical features within the body including the muscles, organs, bones and connective tissues) and, on the other hand, more formal visual descriptions of anatomy and pathology used within medicine. Against this theoretical background, we next present data on individuals’ use of such images and conclude with some implications for practice.

### Understandings of pathophysiology

Lay knowledge is informed by a natural human desire to make the strange familiar [12] and to understand the source of unwelcome feelings – in illness, “identifying the cause of the body’s discomfort becomes imperative” [13, p.131]. Lay understandings can be internally consistent and rational, while changing in the light of new experiences and the availability of believable information [14]. As Cassell [15, p.143] suggests, “it seems quite natural that a person’s use of language reflects his beliefs about disease.”

Throughout the development of modern medicine, the spatial aspects of illness and its location within the body have been an important part of clinical investigation, allowing the body to become “legible” to the clinician’s observing eye [16,17]. The ability of clinicians to locate illness within the body depends on a “considerable visual literacy” [18, p.8] and intimate knowledge of human anatomy. The “underlying spatialization of illness” and the logic of clinical practice and investigation assert that experience and illness are “linked through surface and depth” [17, p.395]. With the discovery of X-rays in 1895 [19], the medical gaze first turned inwards, giving birth to the radiological aphorism “one look is worth a thousand listens” [20, p.340]. Since then, a multitude of imaging modalities – such as fluoroscopy, magnetic resonance imaging, computer tomography scans, ultrasonography images, arthroscopy, endoscopy and laparoscopy [21] – have ensured that vision in medical investigation and diagnosis has become increasingly dominant over time [18, p.8].

At a more fundamental level, we suggest there is often an instinctual desire to *understand* anatomy, to *see* and to *show* what is happening within the body. This desire to understand both the “surface and depth” of the body can be found in the rock art of Australian Aboriginals who documented the internal anatomy and structures of living animals and human beings in “X-ray” style rock paintings as far back as 2000BC, or even earlier [22,23]. The desire to understand what is happening within the body is also present when painful sensations occur, as in disease. Pain focuses consciousness on what is otherwise an absent [24] or a transparent [25] body – one that is taken for granted and of which we are only tacitly aware. Leder [24, p.75] suggests that: “Pain tends to induce self-reflection and isolation. It effects a spatiotemporal constriction […] Our attention is drawn back not only to our own bodies but often to a particular body part.”

While some people seek to communicate their painful condition to others to gain social legitimation, secure help and possibly redemption [26], others visualize, imagine or depict their painful condition to objectify their pain and to complete their own understanding, or to provide an external expression of their internal worlds [27]. In the process, individuals may call upon both experiences from their everyday life-world and more formal referents drawn from biomedical discourse [28].

Thus, individuals objectify pain to make sense of it, to orientate themselves towards its origins, and ultimately to expel unpleasant feelings. Disease and symptoms may be depersonalized and objectified as “the” or “it”, as something distinct from – and often inimical to – the self, suggesting a reflection on the mind-body relationship [15,24,29–31]. As Leder [29, p.262] puts it: “Whereas in day-to-day events we are our body without hesitation, suddenly pain renders the body disharmonious with the self”. There is consequently an attempt to make sense of one’s experience first to oneself and then to others. Lay interpretations and descriptions of pain and disease indicate a struggle to familiarize sensations, to make sense of the body when it no longer functions as before, to give meaning to these events [32,33]. As Leder [24, p.78] suggests, “Pain exerts a telic [purposeful] demand upon us”, which gives rise to a search for interpretation and understanding, “where the body becomes the object of an ongoing interpretive quest”. Leder [24] gives the example of an injured tennis player who stops the game, not just out of an inability to continue, but to seek the origin, extent, and significance of the pain, so that he or she may then be able to take reparatory action or to cope with the existential challenge of pain.

### The clinical context

Patients’ understandings of their condition and pathophysiology are known to influence their response to their illness: whether they consult again, and whether and how they act upon recommended treatment [34–37]. Research shows that there is often discordance between patients’ interpretations of illness and those of health care practitioners (HCPs), with HCPs sometimes perceiving patients to be noncompliant, unintelligent and lazy, while patients can be left confused and dissatisfied with the information they are given [14,38,39]. Equally, patients and HCPs may interpret language very differently, even if the vocabulary they use is shared [40]. Around one-fifth of older people will develop interfering musculoskeletal pain over the course of a 3-year period [41], and chronic musculoskeletal pain, such as from osteoarthritis (OA), is a leading cause of persistent pain and years lived with disability [42]. Although OA is common, a concordance should not be assumed between patients’ and HCPs’ understanding of the disease [14,39]. Differences in how HCPs and patients understand musculoskeletal disease may be a contributing reason for the poor uptake of evidence-based guidelines for the management of OA and suboptimal self-management [43].

Therefore, exploring how people think about their pathophysiology and communicate these insights through images or visualizations can potentially enrich an understanding of the subjective experience of chronic pain and could usefully inform how patients with painful musculoskeletal conditions can be better supported to manage their condition [44].

### Research questions

This paper explores the experiences of older adults (45 and over) in relation to musculoskeletal pathophysiology, mainly OA. Drawing on qualitative data from interviews with patients who have musculoskeletal pain, we explore their interpretations of their pathophysiology, their accounts of HCPs’ explanations of their condition, and HCPs’ use of images and visual models (medical or otherwise) in their explanations. We examine the utility and medical accuracy of patients’ explanations, asking which matters more and to whom, in order to build a perspective on the role of visual and verbal explanations in collaborative, patient-centered practice and suggest how consultations around musculoskeletal disease might be improved through the use of medical images and visual models. We were interested to explore this idea further in the context of musculoskeletal pain, having identified it as a theme in the research studies on which this paper is based.

## Methodology

This paper is a supra-analysis of data from two broader studies. A supra-analysis is defined by Heaton [45] as secondary analysis of qualitative data from a study that transcends the original focus of that study and yet is carried out by members of the original research team.

Study 1 investigated older adults’ experiences of interference from chronic pain in later life [46,47]. The study used semi-structured interviews to explore older adults’ (aged 55 and over) experiences of different pain states, with the aim of examining how older people can best be helped to age well in the presence of musculoskeletal pain. A purposive sample of 60 people was selected using maximum variation sampling [48]. A full account of the methods for this study is provided elsewhere [46,47]. Study 2 was a nested qualitative study within a large clinical trial (Benefits of Exercise for Knee Pain: ISRCTN 93634563) that compared three physiotherapy-led exercise interventions. The aim of the trial was to improve the effectiveness of physiotherapy-based exercise interventions in primary care patients with OA when compared to usual physiotherapy care. Semi-structured qualitative interviews were conducted with 30 adults aged 45 and over. The methods of this study are detailed elsewhere [49,50]. In keeping with Heaton’s [45] definition of a supra-analysis, at least one author (AJM) collected data and contributed to the qualitative analysis for both studies. Both studies received ethical approval from UK National Health Service (NHS) research ethics committees (Study 1: 09/H1203/84 and Study 2: 10/H1017/45).

### Analysis

For the supra-analysis, data from both studies that pertained to participants’ explanations and understanding of their condition were collated and subjected to case-by-case analysis. We noted the language that people used to describe the pathophysiology of their painful joints and any references to imagined physical and topographical features or processes that served to explain or ascribe meaning to their symptoms. Each case was discussed between the authors to ensure a consensus was reached upon the interpretation of these references. While such references were not common to all participants (nor directly elicited within the initial interview schedule), they appeared to be indicative of how a proportion of the participants from both studies understood their pathophysiology. Further details of the primary analyses for the original studies are provided elsewhere [46,47,49,50].

## Findings

While chronic joint pain was the focus of the first study, the second study focused on evaluating a physiotherapy-led intervention for painful knee OA. Both sets of participants, however, offered understandings that often fused information offered by HCPs with their own lay understandings and sensory experiences. Participants’ data are presented in the form of cases that exemplify a range of perspectives and experiences, which include: discordance between patient and clinicians’ understandings of chronic joint pain; the complexity of patient interpretations of joint pain; the orienting effect of medical images and diagrams; patients’ struggles to combine medical and lay knowledge; and combining verbal and visual explanations for a more embodied understanding of joint pain. Pseudonyms are used throughout. Within illustrative quotations, an ellipsis represents a pause. An ellipsis in square brackets represents an omission of speech that is repetitive or that is unrelated to the topic.

## Cases from study 1

### Arthur: discordant images

Discordance can exist between the patient’s experience of illness and the general practitioner’s explanation, giving cause for the patient to (re)interpret events and to relay an alternative interpretation of the diagnosis, regardless of the presence of other medical visual evidence such as X-rays:

I’ve never seen anybody with a neck like mine – stiff. ‘Cause I look around for people that have got and I never see anybody and that, never seen one in [place name] and I go the doctors, ‘yes all right I’ll send you for an X-ray’ and I’ve been for a few on me neck. Well on everything. And I go back to the doctors and ‘Oh yes, you’ve got a bit of wear and tear’ [laughing] and I say ‘Wear and tear? I can’t turn my bloody neck!’

Arthur found a diagnosis of “wear and tear” unacceptable, contrasting this diagnosis with his own perception that he had a very rare type of symptom. On the basis of physical and audible sensations (crunches and cracks), he imagines what is happening inside his body:

I think all the cartilage in the bones has gone [laughing] and I’m down to bare bones, and I don’t know whether that makes it better. Me neck, the spine, the spinal bones must be just rubbing next to one another, ‘cause when I, when I turn me neck and… crunches and cracks and to me that’s bone rubbing together.

Arthur can only communicate verbally what he thinks is happening, based on bodily sensations and feedback such as the sounds and feelings of “crunches” and “cracks”. He imagines the bones rubbing against one another. Regardless of whether or not this corresponds to biological reality, he felt his experience was inconsistent with the wear-and-tear explanation given by the doctor. Specifically, although the term “wear and tear” was not conceptually incompatible in medical terms with Arthur’s perspective on the changes that had occurred in his spine, it appeared to misrepresent what he imagined was the extent and severity of these changes. The everyday, almost banal, connotations of ‘wear and tear’ were at variance with what he saw as the exceptional nature of his symptoms.

### Ray: a “simplistic mind” – or a complex diagnosis?

Ray hypothesized about an ankle injury that had deteriorated over the years, leaving him with increasingly debilitating symptoms. He imagined what had happened inside his body and the effects of this:

I suppose what happened when it healed, presumably it was a, a tendon or a junction with a, with a muscle, it, it pulled and it had… when it had finally healed it was a bit longer than it was before, that’s how my simplistic mind thinks of it, and allowed it to turn over.

While he excused his explanation as simplistic it is, nevertheless, based on a complex mix of sensory feedback, functional capacity, theoretical biomechanics and his attempts to imagine, in the absence of medical knowledge and internal visual images, the intracorporeal space, enabling him to familiarize his symptoms [12]. The disruptions that he imagined within this “internal world” [27] form part of a sophisticated causal account of his symptoms, one that has a logical coherence equal to that of a formal biomedical explanation.

### Greg: a medically-informed orientation to pain

In this and the following example of participants’ orientations to their pathophysiology taken from the first study, there was evidence that HCPs had provided visual explanations of the patients’ pathophysiology, and that this had informed their understanding of their condition and the resulting symptoms.

During his interview, Greg – a former hospital porter – explained that the surgeon gave him a CD containing still images from his arthroscopy:

Greg: I went in again for another arthroscopy, there was absolutely nothing there, just basically bone against bone which he quite happily gave me a CD of… he give us a CD with the actual images… They take stills inside you… showed you the damage that’s in there…

Interviewer: Did that give you a clearer idea of what was going on?

Greg: Yeah, yeah. They do – he does it for a lot of the patients up there [private hospital]… so you can see actually what your knee is like.

Interviewer: Yeah, how does it make you feel when you see those images?

Greg: Well you appreciate where the pain is coming from then. Yeah, it’s not somebody just telling you something, you’ve actually got an image there in front of you.

Interviewer: Does it help you to understand it more?

Greg: Yeah, I think so, yeah. Probably doesn’t suit everybody. Some people don’t like to know, you know, they just want the pain taken away. But I suppose with working in that environment for 16, 17 years, you know, you just get used to it, you know, and it’s – so you know I’ve watched knee replacements and that taking place, so you know what’s… and it’s just interesting to, you know, that’s mine, you know. That’s what it looks like and that’s where I’m getting the pain from.

Interviewer: Yeah. You say it helps you to understand where it’s coming from. It’s like orientating yourself [Greg: yeah, yeah] in terms of what’s wrong with [Greg: yeah]… so it clears up any confusion about why it’s happening or…?

Greg: Yes, I think so. Obviously, you know, it perhaps doesn’t suit everybody… But I found it interesting and – and able to – that’s why I can say yes, that’s causing me the problem.

Arthroscopic images and X-rays of his joint replacement helped Greg to better understand the reason for his pain and its origin. While Greg acknowledges that seeing direct visual evidence of the pathophysiology of one’s body may not suit everybody, he was able to say with certainty that there was “bone against bone” and that he had seen the “damage”. Being able to see the image himself seemed to give Greg a fuller interpretation of an otherwise invisible body [24], and a more collaborative role in the diagnostic process, meaning that he could orientate himself towards the origin and location of his pain, mapping its source within his own intracorporeal space. Thus, in this instance the pain is understood and clearly embodied in terms of images that directly represent biomedical ‘reality’.

### William: drawing diagrams

During the interview, William drew diagrams of his pathology for the interviewer – as his doctor had for him – in order to visually explain his shoulder pain:

I had loads of people to pin-point the problem on me. Poke here, poke there, to try and diagnose what it was. Because I didn’t know, I didn’t know I’d got arthritis until [the consultant] cut me open, and had the X-rays… and said, that it was … He showed me like you know how the arthritis had formed. But it wasn’t anywhere else.

William noted that it was the X-rays and the surgeon that *showed* him how the arthritis had formed, suggesting that these visual elements served to “pin-point” the problem for him. This knowledge allowed him to redraw his pathophysiology in the interview. Essentially, the visualization was provided for him, creating a distinctively medical framework for his own intracorporeal landscape. It made an obvious impression on him, as he recalled what was happening beneath the skin and drew diagrams to show how the surgeon had removed bony nodules around the joint:

William: Oh that’s what had happened […] The two bones, there’s two bones in your shoulder right, the arthritis had grown on the bone there [pointing to shoulder].

Interviewer: Right.

William: Can I draw it again [to] show you?

Interviewer: Yes.

William: [Drawing] I remember, I remember [doctor] telling me. The arthritis had grown here, on that bone there and that one, they’d grown and this tendon came round and that somehow rubbed and snapped it.

Interviewer: I get you, yeah. So, as it had grown it had kind of worn [William: yeah] the tendon out?

William: This, it had grown, the arthritis had grown there and grown along there, it had ripped through that [the tendon] that where it… then that had happened the other side… So what they did when they opened me up they chipped a bit of me arthritis away […] He chipped it all out and pulled them back [the tendons] and stitched it up.

In re-drawing the diagram, William mapped out the features of his intracorporeal landscape and the events that occurred therein, enlisting both a visual and a verbal explanation, allowing him an objective representation of his experience of his condition in a way that he found helpful.

William’s and Greg’s access to images derived directly from biomedical “reality” provided them with a representational landscape. In both cases, this recreated landscape made their symptoms more comprehensible to themselves and more communicable to others, facilitating a visually informed understanding of their condition. Images can therefore serve to assist the individual’s own understanding (as with Greg) and to communicate an explanation to others (as with William).

## Cases from study 2

In the second study, patients received treatment from NHS physiotherapists for their knee OA. In some instances, explanations were accompanied by a model or visual aid, but as the use of such aids is not part of standard care, this was at the discretion of each physiotherapist. In the absence of a model or illustration similar to those described by participants in the first study, those in the second study imagined what was happening within their joint when prompted by audible or visual sensory feedback or physical sensations.

### Gordon: “it would just make you stop and think”

Like Ray and Arthur, Gordon also struggled to understand the origins of the noise and grating sensations within his knee:

Gordon: It’s one of those things where you know, it sounds a lot worse than it actually is at the moment you know, it’s – because you think, is that grating, wearing something? But it doesn’t appear to be, it just appears as though there’s something in there that’s moving about and making the noise, you know, so…

Interviewer: Yeah. That’s something I’m interested in, how people imagine the inside of the knee when they hear sounds like that.

Gordon: Yeah, yeah, it is a funny sound. I don’t know how it does it, I don’t know. I was listening to something on the telly the other day and the guy was saying that when we crack our fingers and it [claps] does that, that that’s a fluid bag inside your finger that’s popping or something, and I think, ‘How the hell’s that? That sounds to me like it’s, like there’s a bone moving against a bone or something,’ […] So I suppose if you did understand a little bit more, perhaps you know, you can do the right things with it rather than, well, it would just make you stop and think, you know.

Gordon imagined that the grating sensation in his knee might have been something “wearing”, but as his knee was not deteriorating he reasoned it was something moving around inside. Acknowledging that his interpretation of the sounds and sensations he experienced might be wrong, he referred to a television program that he had watched, which explained cavitation – a crack sometimes heard with a decrease in intracapsular pressure [51] – which he suggests sounded like “bone moving against bone”. Given the dissonance between the program presenter’s explanation and his own uncertainty about the “funny sound” in his knee, Gordon reasoned that a better understanding of what was happening could help him to “do the right things” and better manage his condition. For Gordon, visualization of his intracorporeal space appears to serve as a heuristic function, as he seeks to reconcile his own understanding of his body with more formal, ‘external’ explanations, testing the former against the latter.

### Jack: showing and knowing

In this and the following case, the physiotherapist used visual aids and models to explain the knee joint and the purpose of the exercises. Jack found that when the physiotherapist used a model of the knee in conjunction with a verbal explanation, this helped him to understand the pathophysiology of the knee and what was happening inside his knee joint:

Jack: And the fact that he knew… he explained me knee joint and he showed it me and he just went through it completely and…

Interviewer: Did that make a difference?

Jack: Yeah certainly, yeah.

Interviewer: In what way?

Jack: ‘Cause you don’t really, I mean I don’t know what’s inside me knee and all that, I mean I’m not a doctor or a physiotherapist and he explained everything to me, what was happening and why it was happening you know.

Interviewer: So how did that make you feel when he explained everything that was going on in your knee?

Jack: I think once you know what’s going on inside that you know what you’ve got to do to put that right, if you like, and why it is, it’s, I mean I know I’m just getting old aren’t I really but, he just explained why it happens and that puts you at ease a little, because you never know what it is do you, really, any pain and once he shows you well that’s worn and that’s because…and it just, you just know what’s going on in your body then, well in your knee, yeah.

Interviewer: Does that mean that the exercises made more sense to you then?

Jack: Yes, yeah, I think they do, yeah.

Just as Gordon hypothesized that a better understanding of what was happening inside his knee would enable him to manage it better, Jack explained that when a verbal explanation was supplemented by the physiotherapist showing him what was happening inside his knee, he then had a better understanding not only of the cause, but also of what he had to do to put it right, and how the exercises would help – and this put him “at ease”. Through this visual explanation, the ‘felt’ and the ‘seen’ were brought together [52] to provide a fuller understanding, and on this basis he was able to plan practical action in relation to his pain.

### Margaret: making sense

Margaret explained how the physiotherapist had used a model of the knee to illustrate the consequences of not adhering to his advice to stop wearing high-heeled footwear. She suggested that this mode of explanation accorded with her desire to know the reason for the advice and how this related to her knee, as she felt a simple verbal explanation would not have convinced her of its value:

Margaret: Yeah, he had models there and things.

Interviewer: So he went through and explained…

Margaret: Yeah, yeah, yeah […] which is what I really liked. I like to know why I’m doing something, and that therefore, made sense to me.

Interviewer: Yeah. Had you had that before?

Margaret: No, because as I say, before, when I went for physio, it was very much them doing it to me and it was an injury. It was, like, ‘Right, you’ve done this, let’s, let’s put it right.’

Interviewer: Hmm. So did that make any difference to you?

Margaret: I think so. I mean I think that’s why some of the stuff stuck with me, you know. If you told me not to wear high heels because it’s vain, I’m not, I’m not going to listen to you, but if you say to me, ‘Look, if you put high heels on,’ and he had the model of the knee, and said, ‘Right, if you, if you raise the back of your leg up here, your knee’s going out like this,’ well, it’s perfectly obvious, isn’t it, to an absolute idiot why you shouldn’t be doing them […] and, yeah, so, so things like that, I think it’s just the way I’m wired, you know. I need to understand why I’m doing it.

For Margaret, the use of a verbal explanation in conjunction with the visual model helped her to understand the rationale for the prescribed intervention and advice and allowed her to understand these in a more embodied manner. Furthermore, by seeing the aberrant biomechanical action caused by the use of high heels, she understood the consequences of wearing them. The visual element in the explanation that Margaret received therefore contributed not so much to her understanding of pain as an experience, as it did to her making sense of what she should do about it.

## Discussion

In both of the reported studies, lay understandings of illness often involved an attempt to describe anatomical features, pathophysiology or related imagery and meet the “telic demand” for understanding that pain exerts [24]. Equally, such visualizations assist in making individuals’ experiences of their symptoms more expressible to others, counteracting to some extent their essentially private and “unshareable” nature [2,3]. They also informed individuals’ current or planned action in relation to their symptoms. Hence, in Leder’s [24] terms, these visualizations have both a *hermeneutic* function, in terms of interpretation and meaning, and a *pragmatic* function, in terms of action.

Frequently, participants had been provided with either verbal information alone or medical images or anatomical models in addition to verbal information. As in the case of Greg, William and Margaret, participants were often able to assimilate this visual information within their own understanding of illness, but for some, such as Arthur, this was only partially achieved, as a gap remained between a medical explanation and his own subjective experience creating a “disarticulation” [13, p.30] or “disharmonious” relationship [29, p.262] between self and body that characterizes pain.

Nonetheless, our analysis suggests that where a verbal explanation was complemented by anatomical models, medical images or illustrations – or where individuals could imaginatively create such visualizations for themselves – they often seemed to gain a new and clearer understanding of their illness. Our resultant hypothesis is that this visually informed understanding can lead patients to a more embodied and objectified orientation to their condition, a greater understanding of the rationale for treatment and prevention recommendations such as physical exercise and involvement in social activities, and thus more incentive to follow such recommendations, as well as greater satisfaction with the HCP’s explanation. It is acknowledged that often patients do not engage with physical exercise, despite its being a core treatment for OA [53]. Using visually informed explanations and anatomical models may help to resolve misunderstandings about the safety of undertaking exercise [54] and thereby facilitate a return to physical activity and social participation.

Considering five paths to meaning – what, when, where, how and why – we suggest that visually facilitated explanations and images show the *what*, *where* and *how* of musculoskeletal symptoms such as pain, stiffness and the consequences of behaviors that are not recommended (e.g., wearing high heels). By showing patients *what* happens, *where* in the body it happens, and *how* it happens, coupled with verbal explanations of *when* it occurs and *why* it causes pain, the use of visual elements can facilitate a new, informed understanding and orientation towards their pathophysiology. Borrowing from generative theory, Bushe [55] asserts that a generative image “allows people [to] see the world anew, identify new options, formulate new strategies, even reform their identity” [55, p.91]. In this sense, the use of generative images by HCPs may facilitate a “collaborative discourse” around pain between patients and HCPs [40, p.254] and be a potential key driver in behavior change for people with chronic painful conditions.

However, it is also important not to discount the images that individuals provide for themselves, which may correspond with varying degrees of accuracy to the anatomical or physiological reality of their medical condition; for example, Arthur’s visualization of his illness did not cohere with a biomedical account. However, if we are in any sense to evaluate whether such accounts are harmful to the patient or present barriers to better self-management, it should be in terms of their utility, rather than their biological accuracy. If the way in which patients visualize their illness allows them to develop effective coping strategies that positively affect their physical or psychological health, these ways of understanding are beneficial, irrespective of their strict biomedical accuracy. As Blaxter [56] notes, lay theories and imagined pathophysiology may not be scientifically accurate, but they are not thereby unscientific. This is exemplified in Ray’s highly developed explanation of his symptoms.

For clinicians, providing an explanation of a painful joint may involve a careful balance between acknowledging the cognitive and practical usefulness of lay understandings, and correcting certain pathophysiological “misunderstandings” (possibly via the use of generative images such as anatomical models) if this may protect the patient from behavior that is potentially harmful (e.g., resting a joint in a situation where it should be exercised and strengthened or vice versa). As an example, Robertson [57, p.187] cautions against ignoring the potentially harmful belief systems of patients with crepitus, who may avoid exercising:

Hence to evade the belief system of patients with crepitus through lack of interest or knowledge is to fail the patient and leave them vulnerable to fear-avoidant behaviour, which may further compound their initial problem.

Although pathologies may be made visible through medical images and models, providing a frame of reference and a new level at which patients and HCPs can communicate, medical images may not always be available, and some patients find such images “creepy” [58, p.152], as was suggested by Greg. A possible solution to avoiding the discomfort that may result from seeing one’s own body “medicalized” in such a way is to use an image from a text book or internet database to explain the condition.

A limitation of this study is that we have focused on nociceptive pain and sensations associated with OA, as reflected in the data. It is important to acknowledge that neuropathic pain and central sensitization may also be present in later-stage OA [59–61]. We recognize that for patients with neuropathic pain a different approach may be required, employing explanations of neurophysiology, as medical images of the structural features of OA, and accounts framed in anatomical or mechanical terms, may not resonate with these patients’ experience of their pain and may therefore not be helpful. However, even for neuropathic pain, the judicious use of visual representations and models of neurophysiology may have a role to play in facilitating patients’ understanding.

We suggest that this study paves the way for further theoretical enquiry and additional empirical consideration of the potential practical implications of the use of visual models and medical images to inform and support patients’ understanding and management of their condition [40,62,63]. A greater understanding of how patients orientate themselves toward their condition in the absence of medical images might also help to improve HCPs’ understanding of patients’ beliefs, attitudes and behavior regarding their condition. We also acknowledge that the use of more visually informed explanations may be more effective for some patients than others, especially those who have a stronger preference for visual learning and more visuospatial intelligence [64,65]. Future studies could usefully look at patient orientations toward other chronic conditions in which features and processes within the intracorporeal landscape have an important bearing on patients’ perception and understanding of their pathophysiology, such as cardiovascular disease or chronic obstructive pulmonary disease.

## Conclusions

Based on the supra-analysis of these data, we may hypothesize that an individual’s orientation towards his or her pathophysiology plays an important role in making sense of a painful condition and in decision making regarding both self-management and professional care. While the utility of a patient’s belief system is not dependent on its medical accuracy, it might be helpful for HCPs to explore patients’ understandings of their pathophysiology; this may provide valuable insights into how individuals understand and act upon their symptoms, and an opportunity to tailor clinical management and correct any misunderstandings that may adversely affect their symptoms.
